# Balloon Pulmonary Angioplasty for Chronic Thromboembolic Pulmonary Hypertension: Identifying and Managing Complications for the Operator

**DOI:** 10.1016/j.jscai.2025.104049

**Published:** 2025-11-13

**Authors:** Jason B. Katz, Kelley Chen, Michael J. Cuttica, Ruben Mylvaganam, Yasmin Raza, Maanasi Samant, Jordan Durkin, Kevin Jin, Benjamin L. Magod, Charles Logan, Benjamin H. Freed, S. Christopher Malaisrie, Stephen F. Chiu, Daniel R. Schimmel

**Affiliations:** a Department of Internal Medicine, Northwestern University Feinberg School of Medicine, Northwestern Memorial Hospital, Chicago, Illinois; b Division of Cardiology, Department of Medicine, Northwestern University Feinberg School of Medicine, Northwestern Memorial Hospital, Chicago, Illinois; c Division of Pulmonary & Critical Care, Department of Medicine, Northwestern University Feinberg School of Medicine, Northwestern Memorial Hospital, Chicago, Illinois; d Northwestern University Feinberg School of Medicine, Chicago, Illinois; e Division of Thoracic Surgery, Department of Surgery, Northwestern University Feinberg School of Medicine, Chicago, Illinois; f Division of Cardiac Surgery, Department of Surgery, Northwestern University Feinberg School of Medicine, Bluhm Cardiovascular Institute, Northwestern Memorial Hospital, Chicago, Illinois

**Keywords:** balloon pulmonary angioplasty, chronic thromboembolic pulmonary hypertension, pulmonary hypertension

## Abstract

Balloon pulmonary angioplasty (BPA) is the treatment of choice for patients with chronic thromboembolic pulmonary hypertension who cannot receive pulmonary thromboendarterectomy or who have residual pulmonary hypertension after pulmonary thromboendarterectomy. As BPA volume and expertise have increased, significant strides in refining this technique have improved success and minimized complications; yet, minimal literature exists on appropriate complication management. This review summarizes how interventionalists can prevent, recognize, and manage BPA complications effectively. Operators must first focus on appropriate patient and vessel selection. Lower lung zones coupled with ring-like stenoses and web lesions are more favorable. Increased patient age, tortuous or subtotal lesions, and elevated pulmonary vascular resistance (>6 Wood units) portend a higher risk for complications. Vascular perforation can be treated with balloon tamponade, embolization (gelfoam and/or coils), and covered stents. Massive hemoptysis is managed with intubation and single lung ventilation if needed. As shock is predominantly hemorrhagic and/or cardiogenic, first-line therapies include vasopressors, inodilators, pulmonary vasodilators, and volume resuscitation, whereas mechanical support devices are second-line. Although reperfusion lung injury (RLI) is uncommon, it is generally well tolerated and managed with positive pressure ventilation, high-flow oxygen therapy, and diuresis. Acute kidney injury can be reduced by dilution of contrast dye, use of extension catheters, and procedural planning. Utilization of standard, angle-minimizing techniques, avoidance of high-dose cinefluoroscopy, and digital subtraction angiography minimizes radiation dose. In conclusion, most complications can be avoided or managed effectively without major morbidity or mortality by optimal preparation, patient selection, and commitment to a comprehensive BPA program.

## Introduction

Chronic thromboembolic pulmonary hypertension (CTEPH) is caused by persistent organized obstruction of the pulmonary arteries, creating a cascade of deleterious consequences, namely, increased right ventricular afterload due to augmented pulmonary vascular resistance (PVR), decreased forward flow (cardiac output), volume overload, and end-organ damage.^1^ Although the treatment of choice for CTEPH is pulmonary thromboendarterectomy (PTE), balloon pulmonary angioplasty (BPA) is recommended for patients who either are not operative candidates or have residual or recurrent pulmonary hypertension (PH) (after PTE and/or PH-targeted medications).^2^

Although initial reports of BPA for inoperable CTEPH demonstrated promising hemodynamic outcomes, there was an unacceptably high incidence of reperfusion pulmonary edema^3^ and resultant respiratory failure. As global BPA volume and expertise have increased, significant strides in technique refinement have decreased complication rates. In most modern single-center experience series and registry data, BPA is now reported as a safe and effective therapy (after an initial learning curve).^4^, ^5^, ^6^ In hopes of helping operators with refining their skill set and approach, this review highlights anticipation, management, and reduction of BPA complications (see Central Illustration).

**Central Illustration:**
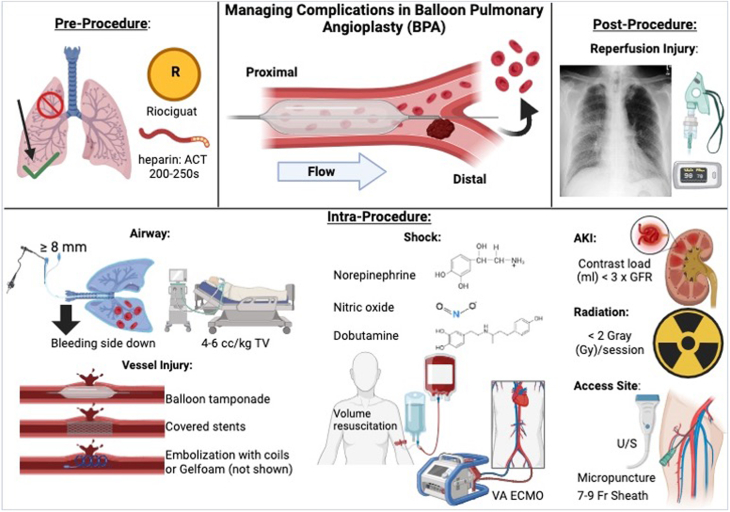
**Listed are the major preprocedural, intraprocedural, and postprocedural considerations when performing balloon pulmonary angioplasty (BPA) and managing complications pertaining to wire perforation, bleeding, and reperfusion pulmonary edema.** ACT, activated clotting time; AKI, acute kidney injury; GFR, glomerular filtration rate; TV, tidal volume; U/S, ultrasound; VA ECMO, venoarterial extracorporeal membrane oxygenation.

## Preprocedure preparation

### Patient selection

Risk mitigation begins with optimizing patient selection. Appropriate patient identification is ideally performed by a multidisciplinary team, consisting of an experienced surgeon, BPA operator, PH physician, and radiologist. The main roles of the team, operating at a comprehensive CTEPH center, are to confirm the diagnosis of CTEPH and formulate the optimal management strategy, including judging the feasibility and anticipated benefit of BPA.

For the BPA operator, special attention should be paid to both absolute and relative procedural contraindications. Severe iodine contrast allergy refractory to premedications like steroids or antihistamines may warrant allergy consultation. Other major contraindications include the inability to remain supine or cooperate with breath holding, access site, or uncontrolled systemic infection, and severe multiorgan dysfunction.^7^^,^^8^ Nevertheless, case reports have been described of successful BPA salvage procedures for patients with accompanying hepatic failure, refractory respiratory infection,^9^ as well as severe, concomitant right heart, hepatic, and renal failure.^10^ Additional patient characteristics resulting in a poor risk-benefit ratio with BPA include comorbid, untreated left heart disease (manifested by high left-ventricular end diastolic or pulmonary artery wedge pressure), severe parenchymal lung disease (particularly in the subtending vascular distributions of anticipated treatment), and coagulopathy.

Other nonmodifiable patient factors, although not absolute contraindications, can elevate risk: advanced age (>75 years), elevated mean pulmonary artery pressure (mPAP) or PVR,^6^ BPA after PTE,^11^ and certain lesion characteristics such as tortuous and subtotal occlusions. A higher percentage of ring-like stenoses and web lesions, along with a lower incidence of total occlusions and tortuous vessels, may portend a greater likelihood of complication-free, successful treatment.^12^^,^^13^ Table 1^6^, ^7^, ^8^^,^^11^^,^^13^ summarizes the above considerations in patient selection. After careful consideration of the aforementioned factors, our center ultimately turns down a minority of patients for BPA, provided an appropriate benefit-to-risk ratio is present.

**Table 1:** BPA patient selection for CTEPH: absolute and relative contraindications along with risk factors for complications.

Absolute contraindication	Relative contraindication	Risk factors for complications^a^
Severe anaphylactic allergic reaction to iodine contrast media despite corticosteroid premedication	Severe chronic or acute single-organ dysfunction^b^^,^^c^ (ie, chronic liver disease, chronic kidney disease)	Age >75 years (excluded from trial^b^ and associated with increased complications^d^)
Inability to lie flat,^b^^,^^c^ undergo right heart catheterization, or cooperate with breath holding	Thrombocytopenia <50,000 per microliter INR >2^c^	Elevated mean pulmonary artery pressure >30 mm Hg^d^ or pulmonary vascular resistance >6 Woods unit^e^
Active severe pulmonary infection, access site skin infection or any uncontrolled infection including bacteremia/fungemia^b^^,^^c^	Mild to moderate infection	CTEPH lesioncharacteristics^f^: Tortuous vessels (high complication rate) Subtotal occlusions (high complication rate) Chronic total occlusions (low successful intervention rate)
Acute multiorgan failure^b^ (ie, liver and renal failure)	Uncontrolled asymptomatic hypertension, diabetes, or COPD^c^	Residual pulmonary hypertension after PTE^d^
Active hemorrhage^b^	Severe parenchymal lung disease in the vascular distributions of anticipated treatment	–
Acute decompensating right ventricular failure refractory to medical support	–	–

BPA, balloon pulmonary angioplasty; COPD, chronic obstructive pulmonary disease; CTEPH, chronic thromboembolic pulmonary hypertension; INR, international normalized ratio; PTE, pulmonary thromboendarterectomy.

a Despite risk factors, the ultimate decision regarding BPA involves a multidisciplinary approach. Patient-specific considerations include PTE surgery consent, eligibility, and outcome as well as medical optimization with volume management and oral riociguat.

b Adapted from Zhao et al.^7^

c Adapted from Inami et al.^8^

d Adapted from Ito et al.^11^

e Adapted from Wiedenroth et al.^6^

f Adapted from Kawakami et al.^13^

### Preprocedural care

Volume status, cardiac output optimization, and management of anticoagulation are the principal concerns in the preprocedural phase. Right heart catheterization can be used to optimize hemodynamics, with titration of pulmonary vasodilators and diuretics. All PH-targeted medical therapies (oral and/or parenteral) are continued perioperatively. Riociguat in particular has been shown to lower the risk of major adverse BPA effects for patients with PVR >4 WU.^14^ As a mainstay of CTEPH treatment, it is important to balance the risks of holding anticoagulation with the benefit of reducing periprocedural bleeding. Most centers hold anticoagulation during the periprocedural period. Our center typically withholds direct oral anticoagulants 24 hours prior to BPA, whereas warfarin is held 3 to 4 days prior, accompanied by low molecular weight heparin bridging the day prior to the procedure. BPA is only performed if the international normalized ratio is <2.

### Staffing and equipment

The presence of an experienced operator is imperative when performing the procedure, preferably in a high-volume CTEPH center with expertise in both PTE and BPA.^11^ It is well documented that annual complication rates decline with a rising number of procedures,^6^ potentially a reflection of both improved operator expertise and an inherent stepwise reduction in pulmonary artery pressures from prior sessions. There is currently limited volume or skill-based competence criteria for what constitutes an experienced operation. One definition of an “expert BPA center” is >100 procedures per year with mortality <1%, and extracorporeal membrane oxygenation (ECMO) support capability.^15^ Therefore, it is the opinion of our group that an experienced operator functions within the confines of the following criteria which includes: (1) a comprehensive program at a center with a dedicated surgeon with training in PTE, (2) a dedicated pulmonologist or cardiologist who primarily treats PH as their practice, (3) regular meetings for multidisciplinary patient evaluation, and (4) ongoing review of outcomes which include both complication rates and measurements of success as measurement by 6-minute walk and invasive hemodynamic follow-up.

A well-trained anesthesia team is vital to ensure a prompt response to complications involving intubation, single lung ventilation (subsequently discussed in detail), and resuscitation, as needed. Access to interventional pulmonology support for advanced bronchoscopy is also advantageous. ECMO should be available in case of refractory hemodynamic collapse. Essential, specialized equipment for emergencies includes gelatin sponges, covered stents, and vascular coils (see Table 2 for full description).

**Table 2:** Necessary equipment needed for BPA complication management.

Complications	Equipment
Vascular perforation	Tamponade balloon Gelatin sponges Covered stents Vascular embolization coils Blood products Protamine Chest tube kit
Airway compromise - Acute hypoxic/hypercarbic respiratory failure - Loss of patent airway	Large diameter (≥8 mm) endotracheal tube Dual-lumen endotracheal tube Bronchial blockers Suction catheters and canisters × 2 Portable bronchoscopy Oxygen (hi-flow nasal cannula) Noninvasive ventilation (bilevel or continuous positive airway pressure) Invasive mechanical ventilator
Shock	Vasopressors (norepinephrine, vasopressin, epinephrine) Inotropes (dobutamine, milrinone) Pulmonary vasodilators (inhaled nitric oxide, systemic epoprostenol) VA ECMO
Acute kidney injury	Renal replacement therapies (dialysis)
Reperfusion pulmonary edema	Continuous pulse oximetry Serial chest radiographs Oxygen (hi-flow nasal cannula) vs Noninvasive ventilation (bilevel or continuous positive airway pressure) vs Invasive mechanical ventilator

BPA, balloon pulmonary angioplasty; VA ECMO, venoarterial extracorporeal membrane oxygenation.

## Intraprocedural management

Reported procedural complications include access site complications, pulmonary artery injury (including vessel dissection, perforation, and hemorrhage), hemoptysis, and shock. Additional peri-procedural complications comprise acute kidney injury, radiation injury, reperfusion pulmonary edema, and death.^16^ Approximate incidence rates (%) for various complications are included in Table 3.^17^, ^18^, ^19^

**Table 3:** Incidence rates (%) of complications following balloon pulmonary angioplasty.

Complication	Incidence rate (%)
Hemoptysis	7.07^a^-7.7^b^
Pulmonary artery vessel injury	5.05^a^ -7.7^b^
Reperfusion pulmonary edema	1.4^b^-5^c^
Acute kidney injury	0.51^a^
Death	0.31^a^ -0.8^b^
Access site complication	0.21^a^
Infection	0.21^a^
Arrhythmia	0.15^a^
Shock	0^c^

a Adapted from Kennedy et al.^17^

b Adapted from Jain et al.^19^

c Adapted from Ogo et al.^18^

### Access site complications

The femoral vein^18^^,^^20^ is the most common vascular access site for BPA; typical site complications may occur. Earlier complications include inadvertent arterial puncture, hematoma formation, and retroperitoneal hemorrhage, whereas later complications consist of bloodstream infection, phlebitis, thrombosis, erosions/perforations, and aneurysms/pseudoaneurysms.^21^ Furthermore, our practice is to utilize a 9F sheath when performing femoral venous access; a long 7F sheath is then placed in telescoping fashion and may improve patient comfort with guide manipulation. Ultrasound^22^ and fluoroscopic^23^ guidance for micropuncture^24^ venous access are well-described modalities to reduce access site complications. Moreover, ultrasound use is imperative in the CTEPH population to rule out access site thrombus. The operator should select an interventional guide and sheath of suitable size to accommodate the largest balloon to be employed for a targeted vascular territory.

Although not an access complication in itself, femoral vein access may cause difficulty during a right upper lobe intervention, with right internal jugular vein access preferred. In our experience, for some patients with a very dilated left pulmonary artery, when pushing the sheath and guide from the femoral venous access point, the motion of the tools will begin to rise into the apex of the left pulmonary artery. This will cause the sheath and guide to be angled downward when intervening on the right lung and can make intubation of the right truncus anterior complicated, depending on its anatomical takeoff. Because of this catheter behavior, right internal jugular access may be preferred when intervening in the right truncus anterior.

### Pulmonary artery injury

#### Vessel selection

**Figure 1:**
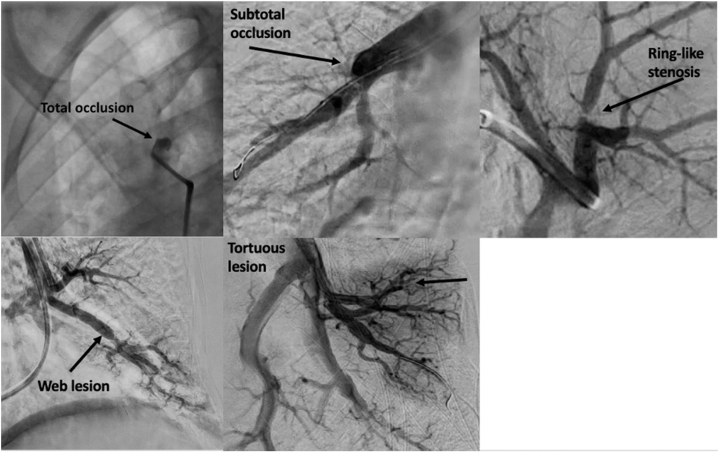
**Representation of various types of lesions commonly encountered on pulmonary angiogram for patients with chronic thromboembolic pulmonary hypertension: total, subtotal, ring-like, web, and tortuous**.

Lesion location and characteristics vary, with different appearances on angiography (Figure 1). Risk/benefit assessment is always the foremost consideration with lesion selection. In our practice, we target lower lobe lesions first, given the greatest hemodynamic benefit; the preferential blood flow confers the utmost reduction in pulmonary pressures.^12^ In addition, ring-like stenoses and web lesions are more likely to be amenable to successful intervention, with a reduced incidence of wire-related complications.

On the other hand, treatment of tortuous lesions and total occlusions tends to be less successful, with a higher incidence of complications. In one study, comprising 97 patients (totaling 1936 interventions), 48% (total occlusions) and 36% (tortuous lesions)^13^ were unsuccessful. Furthermore, the highest complication rate occurred with tortuous lesions (40%), with subtotal lesion intervention leading to the second highest complication rate. Complications from tortuous lesions derive from the lack of adequate wire control, particularly during the respiratory cycle, or from straightening a noncompliant vessel with balloon inflation. Alternatively, total occlusions, web lesions, and ring-like stenoses all had a complication rate of less than 6% in this cohort. The authors postulate that the low complication rates with chronic total occlusion intervention are often due to the inability to pass the wire, thus lessening the opportunity for wire-induced perforation.

Studies from Japan and Germany suggest that utilization of cone-beam and electrocardiogram-gated computed tomography (CT) more accurately identifies distal CTEPH lesions, resulting in improved intervention outcomes.^18^^,^^25^ However, contrast use with CT imaging can increase the risk of contrast-induced nephropathy. Complementary intraoperative imaging guidance via intravascular ultrasound (IVUS)^26^ and optical coherence tomography (OCT)^27^ can prevent balloon oversizing and subsequent vascular injury. Nevertheless, IVUS is utilized far more to augment BPA compared to OCT because it does not require complete contrast opacification of the vessel, thereby reducing both total contrast volume and risk of kidney injury. The injection of contrast medium at high pressure can increase the risk of pulmonary hemorrhage.^11^ This may sway the operator to forgo OCT as a preoperative vessel imaging modality, as it requires a high pressure contrast injection.^27^ Lastly, care must be taken to avoid iatrogenic vascular injury from the imaging equipment itself.

#### Catheter and wire selection/manipulation

Common catheters to safely perform pulmonary angiograms for BPA procedures include Berman catheters, angled pigtail catheters, and APC or other shaped pulmonary catheters.^28^ Subselective cannulation can be performed with coronary guide catheters (ie, Judkins Right 4 cm, multipurpose catheter, etc.) in conjunction with guide extension catheters.^29^ French size will be impacted by the size of the treated vessel. Any vessel of 6 mm or more will benefit from a larger-sized guide for ease of exchanging balloons. Planning for potential complications and choosing adequately large and stable devices to address possible vessel injury is an important dimension of case planning.

A pressure catheter, pressure wire, or, in specific situations, deep engagement of a guide extension catheter, can be utilized to allow for real-time evaluation of prestenotic/poststenotic pressure gradients to identify lesions for therapy and assess the effect of intervention. These can be particularly useful when a discrepancy exists between normal-appearing vessels on selective pulmonary angiography and abnormal perfusion on noninvasive lung imaging.^30^ Analogous to the principle of percutaneous coronary functional assessment, a nonhyperemic pressure ratio (ie, resting, without adenosine) can be calculated by determining the distal-to-proximal mPAP ratio relative to the target lesion. If the baseline mPAP centrally is greater than 35 mm Hg, the end point of balloon dilatation distal-to-proximal pressure ratio should be greater than 0.80.^2^ Pressure assessment helps with risk stratifying for complications, identifying lesions for treatment, and assessing effects of treatment. Attempting to normalize the distal-to-proximal pressure ratio to 1.0 and/or raising the distal mPAP to >35 mm Hg can increase the risk of postprocedure lung injury.

Lesion crossing should be achieved with nonhydrophilic^20^ coronary guide wires (ie, Runthrough, Prowater flex, Sion blue)^29^ due to the friable nature of pulmonary arteries.

Avoiding superfluous or oversized motions of the guide wire, particularly for tortuous lesions, is important to prevent vessel injury; even subtle operator or patient-related motions can lead to injury of small vessels.^13^ Additionally, some experts prefer to maintain a loop in the wire distal to tortuous lesions to minimize the chance of branch perforation.^20^

#### Balloon selection and manipulation

Appropriate balloon sizing is critical to minimize the risk of reperfusion lung injury (RLI) and vessel perforation. An undersized, highly compliant balloon, particularly for total occlusion lesions, can be useful to minimize the risk of RLI, particularly if mPAP is >35 mm Hg.^20^ Excessive balloon dilation of pulmonary arteries with extensive chronic obstructive fibrous burden can result in increased shear stress of the vessel wall and risk for extravasation.^31^ Thus, although increased dilation is associated with greater PVR reduction, it may be preferred to underdilate initially, in several different target lesions, rather than risk injury from overdilating a single site. Interval pulmonary angiography of vessels ballooned via an undersized approach often shows lumen “growth” compared to prior, facilitating further angioplasty with larger balloons. Furthermore, some operators propose that the presence of brisk venous return in the supplied segmental territory is the best signifier of adequately sized and safe balloon dilation.^20^ We prefer to use a combination of visual estimation of vessel size and IVUS to accurately size and select balloons.

#### Vessel injury without hemoptysis

Vessel dissection may occur due to catheter or wire manipulation, particularly with total or subtotal occlusions. Typically, it is well tolerated without notable patient symptoms. Occasionally, an occlusion successfully wired in the vessel, but in a dissection plane, may be initially treated with small, low-pressure balloon inflation. Ultimately, following vessel healing, the lesion can be definitively treated in a similar fashion to investment procedures often utilized in the chronic total occlusion percutaneous coronary intervention.

#### Vessel injury with hemoptysis

**Figure 2:**
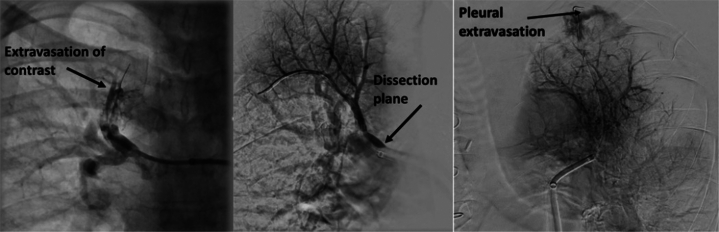
**Pulmonary angiogram showing an unconfined tear (left) in which contrast is seen extravasating into the airway.** Another patient’s pulmonary angiogram (middle), showing a confined tear in the dissection plane. A third patient’s angiogram demonstrating perforation (right) leading to extravasation of contrast into the pleura.

Swift and decisive action following perforation or rupture is required to avoid hypoxic arrest or hemorrhagic shock. Oftentimes, an unexpected course or curling of the wire, with or without a new patient cough, can be an early sign of vessel injury.^6^ The first step in the management of vessel injury is delineating a confined versus an unconfined tear.^32^ With confined tears, contrast extravasation is limited to the immediate peri-vessel area. On the other hand, unconfined tears are characterized by contrast spreading into the ipsilateral lobar and/or pleural space (see Figure 2, rightmost image); in addition, the patient may develop hemodynamic instability and respiratory distress. Extrapolating from the literature regarding pulmonary artery stenosis ballooning, unconfined tears have markedly elevated mortality compared to confined tears.^32^

**Figure 3:**
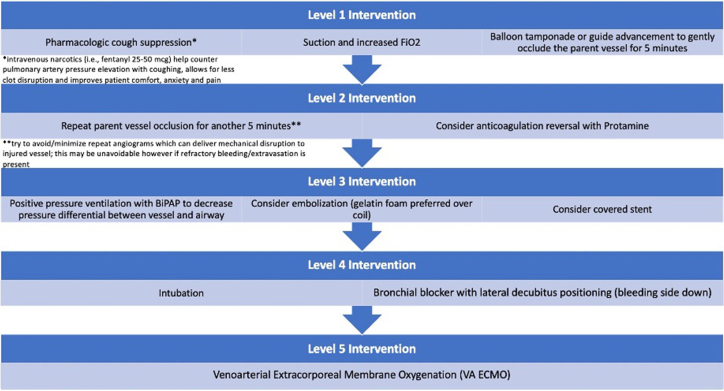
**Stepwise escalation of interventions to manage pulmonary artery injury and hemorrhage during balloon pulmonary angioplasty for chronic thromboembolic pulmonary hypertension.** BiPAP, bilevel positive airway pressure; FiO2, fraction inspired oxygen.

The 3 main options to treat vessel injury with active extravasation include balloon tamponade alone, pulmonary artery embolization (ie, gelfoam and/or coils, with or without balloon tamponade), and the use of a covered stent. Our preferred stepwise approach of interventions is summarized in Figure 3.

The first signal of hemoptysis is cough. Our response to a cough is to pause the procedure and suction to assess for hemoptysis. Should there be bloody sputum, we often utilize angiography to verify the diagnosis and site of extravasation. Our treatment approach is to seat the guide securely to prevent loss of wire access to the vessel during any potential vigorous cough. We then proceed with an initial first attempt at prolonged proximal balloon tamponade, in combination with supplemental oxygen as needed. Should there be angiographic evidence of extravasation, we halt the procedure and perform a 5-minute balloon occlusion. Afterward, we observe for recurrent or worsening hemoptysis, maintaining all wires and catheters in place. For persistent hemoptysis, we first perform a second round of balloon occlusion followed by anticoagulation reversal with protamine if there is continued bleeding; this combination sufficiently controls bleeding in the majority of cases. Although potentially causing the equivalent of a subsegmental acute thrombosis of the injured vessel, it is well tolerated. Of note, balloon occlusion alone can successfully treat wire perforation during interventions for web and slit lesions.^18^ Notably, total occlusions were excluded in this study. In our experience, balloon occlusion may be achieved in the setting of a wire perforation in a total occlusion if the occlusion is midsegment. However, balloon occlusion at the transition of a lobar branch into the ostial segment of a vessel creates technical challenges due to the large parent vessel size.

If hemoptysis persists despite the above measures, we reocclude the vessel with balloon inflation and prepare for embolization. Our first choice for embolization is absorbable gelatin sponges,^11^ given that resorption occurs over time, thereby permitting future attempts at revascularization. Embolization coils are also effective for pulmonary artery perforation related to BPA,^26^ as well as balloon dilation for pulmonary artery stenosis^32^ or suction embolectomy.^33^ Case reports show that covered stents can immediately tamponade extravasation while providing durable patency for years postprocedure.^34^ Nonetheless, covered stents are infrequently used; the most common etiology of bleeding is wire perforation at the distal tip, precluding stent delivery. Moreover, we may use intravenous fentanyl to help suppress severe cough-related clot dislodgment, reducing large spikes in pulmonary artery pressures, and improving patient comfort. Regardless of hemoptysis severity, bloody sputum may persist for 1 to 3 days, and it is often due to clearing preexisting blood products.

In cases of a vessel injury and/or bleeding, conservative measures are usually successful. Nevertheless, although short-term oropharyngeal suctioning, nasal cannula, high-flow oxygen, and/or mask noninvasive ventilation (continuous positive airway pressure/bilevel positive airway pressure)^30^ can be used for milder respiratory compromise, the operator should have a low threshold for escalation to a definitive airway in the setting of massive hemoptysis. Stabilization of the airway includes 3 main protective strategies: standard tracheal intubation (≥8 mm diameter),^35^^,^^36^ with placement of a bronchial blocker, mainstem ventilation of the contralateral (nonintervened upon) lung, or placement of a double-lumen endotracheal tube. Limiting each BPA session to only one lung is critical to allow for prompt unilateral intervention.

Pulmonary artery injury can result in hypoxemia, hypercapnia, or both. No study definitively shows pressure vs volume-controlled ventilation to be superior. We, therefore, present general recommendations below with consideration for individual operator expertise and familiarity with ventilator management.

Ventilator management differs depending on whether single- or dual lung ventilation was established. For a single lung approach, 4 to 6 mL/kg tidal volume should be delivered, versus the often cited 6 to 8 mL/kg during standard dual lung ventilation in acute respiratory distress syndrome. Alveolar hypercapnia and a localized increased PVR in the underventilated lung may improve shunt fraction to the healthy/noninjured. Nonetheless, the benefits of PVR increase must be balanced with its negative effects on right ventricular function in CTEPH patients. Additional ventilation strategies include maintaining plateau inspiratory pressures less than 30 cm H_2_O and positive end-expiratory pressure at 5 to 10 mm Hg.^37^ Inhaled nitric oxide can improve V/Q mismatch and therefore treat refractory hypoxia.

In cases of persistent or severe hemodynamic compromise, femoral-femoral venoarterial (VA) ECMO should be utilized. VA ECMO utilization in post-PTE hemoptysis management has revealed benefits, namely, partial unloading of the right ventricle, decreased flow through the pulmonary arterial tree, thereby reducing pressure on the injured vessel, augmenting perfusion pressure without the use of inotropes or vasopressors and providing oxygenation. In cases of so-called “Harlequin” or “North-South” syndrome, defined by differential oxygenation with risk for cerebral hypoxia, the circuit can be upgraded to V-AV ECMO with the addition of an internal jugular vein return outflow cannula. ECMO utilization can also allow for reversal of anticoagulation and correction of coagulopathy. Indeed, multiple case series have demonstrated the safety of VA or VV ECMO without systemic anticoagulation.^38^^,^^39^ The former study demonstrated that the group without anticoagulation actually had fewer thrombotic complications (13% vs 21%) with statistically nonsignificant differences in overall duration on VA ECMO.

### Acute kidney injury and radiation injury

To prevent acute kidney injury from contrast-induced nephropathy, in our expert opinion, the contrast load should not exceed 3 times the glomerular filtration rate^29^ per session. Although the average total contrast per sessions ranges between 100 to 300 mL, it is typically less than 200 mL at our center. Adequate patient education on breath holding, combined with confirmation of proper catheter placement and avoidance of extraneous imaging, further minimizes contrast utilization. Furthermore, contrast dilution can reduce total volume without significantly compromising resolution. We utilize a 50% diluted contrast with a manifold during our interventions. In addition, extension catheters minimize contrast dose by providing more selective injections. Moreover, leveraging the wire position in complementary angiographic views can identify specific segments without contrast.

To prevent cutaneous radiation injury, the dose should be limited to less than 2 Gy.^20^ Some experts contend that digital subtraction angiography is not routinely recommended in order to minimize total radiation dose.^20^ In our practice, we often use digital subtraction angiography for the identification of target vessels, followed by basic fluoroscopy for the intervention. IVUS can also identify diseased segments without the use of contrast and decrease radiation dosage.

## Postprocedure RLI

Onset of RLI—also referred to as reperfusion edema and/or acute lung injury (ALI)—is usually 24 to 72 hours after BPA, but can be delayed up to 7 days postprocedure.^40^ Although patients with RLI can be asymptomatic, they may present with hypoxemia, foamy secretions, hemoptysis, and/or new infiltrates.^6^ The mechanism of RLI is likely multifactorial, involving vascular stress/injury, endothelial dysfunction with inflammation, and intravascular pressure differentials. A United Kingdom study suggests that ALI is associated with a higher baseline hemodynamic severity (mPAP >50 mm Hg, PVR >9.9) and a greater number of vessels (7.2) treated. Furthermore, treatment of proximal vessels (ie, interlobar artery, basal trunks, etc.) confer increased risk of clinical ALI due to the larger vascular bed subtended. Interestingly, greater symptom improvement along with a decrease in N-terminal pro–B-type natriuretic peptide was seen in patients with either radiographic or clinical evidence of RLI within 2 or 3 days post-BPA.^40^ All patients in this study were treated conservatively with noninvasive oxygen. Consequently, although minimizing patient harm should be the utmost priority, if RLI occurs, it may subsequently be associated with greater procedural benefit.

Post-BPA monitoring is appropriate to ensure adequate hemodynamics and oxygenation. Operators should remain cognizant that patients may initially appear asymptomatic, with reperfusion injury occurring subsequently and unexpectedly.^26^ Our patients remain on continuous pulse oximetry post-BPA until discharge. Patients undergoing their first session and those with risk factors for reperfusion edema are observed overnight in the recovery unit. Patients who have undergone a successful BPA procedure previously without complication, and/or without risk factors for reperfusion edema, may be candidates for same-day discharge.

As mentioned earlier, the use of adjunctive intravascular tools such as pressure wires or microcatheters may minimize the incidence of RLI, as gauging adequate reduction in lesion gradients can prevent overtreatment. Treatment of a distal-to-proximal ratio of greater than 0.80, without attempting to eliminate the gradient, significantly reduces the rate of RLI. Simultaneously, operators should recall that a distal vessel pressure gradient >35 mm Hg post-BPA increases the risk of RLI.

Conversely, evidence to support the routine use of epoprostenol, methylprednisolone, and noninvasive ventilation in RLI prevention is lacking.^31^ Patients should be instructed on the possibility of delayed onset of shortness of breath or cough. They should seek the nearest emergency center for evaluation with a chest x-ray or CT scan and treatment with supplemental oxygen as needed. Ultimately, transfer to the primary BPA center may be necessary. Some BPA operators administer prophylactic intravenous diuresis at the end of the procedure, whereas some only administer if there is an elevated wedge pressure and/or RLI manifests. In patients undergoing PTE, this strategy is accepted practice to reduce the risk of postoperative pulmonary edema and, mechanistically, may have the same benefit in patients undergoing BPA.

## Conclusion

Balloon pulmonary angioplasty is the treatment of choice for patients with CTEPH who cannot receive PTE or who have residual PH after PTE. Most complications can be avoided or managed effectively without major morbidity or mortality if there is adequate preparation, patient selection, and commitment to a comprehensive program with appropriate expertise, facilities, and support.

## Declaration of competing interest

The authors declared no potential conflicts of interest with respect to the research, authorship, and/or publication of this article.
